# Association of Fibrinogen with Severity of Stable Coronary Artery Disease in Patients with Type 2 Diabetic Mellitus

**DOI:** 10.1155/2014/485687

**Published:** 2014-04-06

**Authors:** Li-Feng Hong, Xiao-Lin Li, Song-Hui Luo, Yuan-Lin Guo, Cheng-Gang Zhu, Ping Qing, Na-Qiong Wu, Jian-Jun Li

**Affiliations:** ^1^Division of Dyslipidemia, State Key Laboratory of Cardiovascular Disease, Fu Wai Hospital, National Center for Cardiovascular Diseases, Chinese Academy of Medical Sciences, Peking Union Medical College, BeiLiShi Road 167, Beijing 100037, China; ^2^Department of Cardiology, The Fifth Hospital of Wuhan & Affiliated Guangci Hospital of Wuhan University, Wuhan 430050, China

## Abstract

*Background*. Some studies have suggested a relation of plasma fibrinogen to the severity of coronary artery disease (CAD). However, whether plasma fibrinogen can predict the presence and severity of CAD in patients with diabetes mellitus has not been determined. *Methods*. A total of consecutive 373 diabetic patients with typical angina pectoris who received coronary angiography were enrolled and classified into three groups by tertiles of Gensini score (GS, low group <8; intermediate group 8*~*28; high group >28). The relationship between fibrinogen and GS was evaluated. *Results*. There were correlations of fibrinogen with hemoglobin A1c, C-reactive protein, and GS (*r* = 0.17, *r* = 0.52, and *r* = 0.21, resp.; all *P* < 0.001). Area under the receivers operating characteristic curve of fibrinogen was 0.62 (95% CI 0.56–0.68, *P* < 0.001) for predicting a high GS. Multivariate analysis suggested that plasma fibrinogen was an independent predictor of a high GS for diabetic patients (OR = 1.40, 95% CI 1.04–1.88, and *P* = 0.026) after adjusting for traditional risk factors of CAD. *Conclusions*. The present data indicated that plasma fibrinogen, a readily measurable systematic inflammatory marker, appeared to be an independent predictor for the severity of CAD in diabetic patients.

## 1. Introduction

Several previous studies have suggested a relation of plasma fibrinogen to the severity of coronary artery disease (CAD) [1–6]. Moreover, few of them confirmed a correlation of high circulating plasma fibrinogen with adverse outcome in patients with CAD [1, 4, 6]. Meanwhile, recent observations indicated a relationship between fibrinogen and atherosclerotic plaque progress in patients with acute coronary syndrome (ACS) and stable CAD [7–9].

More interestingly, few investigations suggested that glycohemoglobin might affect plasma fibrinogen concentrations in both genders and circulating high-sensitivity C-reactive protein (hs-CRP) in man [10, 11]. It also has been reported that the patients with HIV infection or unstable CAD companied by persistent chlamydia pneumonia had a higher plasma fibrinogen levels [12, 13], and the higher fibrinogen levels could predict progression of coronary calcification, independent of traditional cardiovascular risk factors [14, 15]. Furthermore, the elevated fibrinogen concentration was independently associated with the risk of ischemic myocardial injury regardless of platelet reactivity by the VerifyNow assay in patients who received percutaneous coronary intervention (PCI) [16]. More recently, a study showed that reduced vagal outflow to the heart was correlated with elevated plasma fibrinogen levels independent of the established cardiovascular risk factors [17]. Psychobiological trials also suggested a dynamic relationship between depression and the plasma fibrinogen concentration which serviced as an inflammatory marker [18, 19].

Based on these studies, plasma fibrinogen appears to be not only an inflammatory marker linking to thrombotic disease but also a predictor connecting with the cardiovascular events. However, whether fibrinogen level has a causal relation to the diseases or reflects genetic variability and residual confounding by other risk factors has been controversial [20–24]. In addition, a small sample size study showed that plasma fibrinogen levels were significantly higher in diabetic patients than that in healthy control [25], suggesting that the fibrinogen might be worthy of further investigation in patients with diabetes mellitus (DM). However, to the best of our knowledge, whether plasma fibrinogen can predict the presence and severity of CAD in patients with DM remains unknown. We, therefore, prospectively examined the relationship between plasma fibrinogen and the severity of CAD in patients with DM who underwent coronary angiography.

## 2. Methods

### 2.1. Study Design and Population

The study complied with the Declaration of Helsinki and was approved by the hospital ethnic review board (Fu Wai Hospital & National Center for Cardiovascular Diseases, Beijing, China). Informed written consent was obtained from all patients enrolled in this analysis.

From June 2011 to March 2012, we prospectively enrolled 373 consecutive women and men (70.2%) who aged from 31 to 79 years (average age 58.7 years) type 2 DM patients with typical stable exertional angina pectoris were referred for selective coronary angiography at our center. Patients with active cardiopulmonary diseases and serious systematic disease such as type 1 DM, acute coronary syndrome, significant hematologic disorders (white blood cell count ≤3.5 × 10^9^/L or ≥20 × 10^9^/L) and/or thrombosis, infectious or inflammatory disease, severe liver and/or renal insufficiency, and various cancers were excluded from the current study. In this study, the detailed demographic, clinical, hematologic, and angiographic data were collected from all subjects.

Hypertension was defined as repeated (at least two times in different peaceful circumstances) blood pressure measurements ≥140/90 mmHg or currently taking antihypertensive drugs. DM was diagnosed in patients with fasting serum glucose level of ≥6.99 mmol/L by multiple determinations or under active treatment with insulin or oral hypoglycemic agents. The hyperlipidemia was defined as low-density lipoprotein cholesterol ≥160 mg/dL and/or triglyceride (TG) ≥200 mg/dL. CAD was defined as the presence of significant obstructive stenosis, at least 50% of the vessel lumen diameters, in any of the main coronary arteries by at least two independent senior interventional cardiologists based on quantity coronary angiography. The severity of CAD was assessed by Gensini score system, which was old but still useful and popular in cardiovascular medicine [26]. The left ventricular ejection fraction (LVEF) was evaluated by echocardiograph using the area-length methods with modified Simpson's rule.

### 2.2. Biomarker Measurements

Venous blood samples were obtained from each patient at baseline upon admission. Plasma concentrations of fibrinogen were measured by using of the Clauss method as previously reported [27]. The levels of hemoglobin A1c (HbA1c) were measured using the Tosoh G7 Automate HPLC Analyzer (TOSOH Bioscience, Japan). The levels of hs-CRP were determined using immunoturbidimetry (Beckmann Assay 360, Bera, California, USA). Total cholesterol and triglyceride were measured by enzymatic methods and high-density lipoprotein cholesterol (HDL-C) by a direct method (Roche Diagnostics, Basel, Switzerland). Low-density lipoprotein cholesterol (LDL-C) was obtained by Friedewald's formula (if fasting triglycerides < 3.39 mmol/L) or by ultracentrifugation. ApoB was measured by an immunoturbidimetric method (Tina-quant, Roche Diagnostics) calibrated against the World Health Organization/International Federation of Clinical Chemistry reference standard SP3-07. All other included biomarkers were analyzed by standard hematological and biochemical tests.

### 2.3. Statistical Analysis

Quantitative variables were expressed as mean ± standard deviation (SD), and qualitative variables were expressed as numbers and percentages. Continuous variables and categorical variables were analyzed by the Kruskal-Wallis test, chi-squared statistic tests, or Student's *t* tests when appropriate. Association between variables was examined using the Spearman and Pearson correlation coefficient, when appropriate. Receivers operating characteristic (ROC) curves were constructed at the most discriminating cut-off values aimed to document the predictive power of plasma fibrinogen for high GS. Based on the tertiles of GS, the enrolled patients were classified into the three groups (low group < 8-point, *n* = 143; intermediate group 8–28 points, *n* = 109; high group > 28-point, *n* = 121). Predictive ability of plasma fibrinogen for high GS (over than 28 points) was carried out by binary logistic regression models using forward stepwise selection process. Interobserver reproducibility was assessed by Bland-Altman analysis and intraobserver reproducibility was assessed by intraclass correlation coefficient. A *P* value of less than 0.05 was considered as statistically significant. Statistical studies were carried out with the SPSS program (version 19.0, SPSS, Chicago, IL, USA).

## 3. Results

### 3.1. Baseline Characteristics

The study population of current observation consisted of 373 diabetic patients referred to coronary angiography with an average age of 58.7 ± 9.6 (ranged from 31 to 79 years) due to typical angina pectoris. The baseline demographic, clinical characteristics and laboratory findings of the enrolled subjects by the tertiles of GS (low group < 8 *n* = 143; intermediate group 8~28, *n* = 109; high group > 28, *n* = 121) were summarized in Table 1. In brief, patients with higher GS were often accompanied with lower LVEF and HDL-C, but higher N-terminal pro-Brain natriuretic peptide (NT-pro-BNP), and HbA1c and fasting blood glucose (FBG). Meanwhile, the major inflammatory and oxidative stress biomarkers such as leucocyte count, uric acid, and hs-CRP among the groups were significantly unbalanced. Specifically, plasma fibrinogen concentrations were significantly different assessed by both trend analysis for the three groups and comparison test for the high GS and low-intermediate GS group.

### 3.2. Correlation between Plasma Fibrinogen and Hemoglobin A1c, hs-CRP, and GS

**Figure 1:**
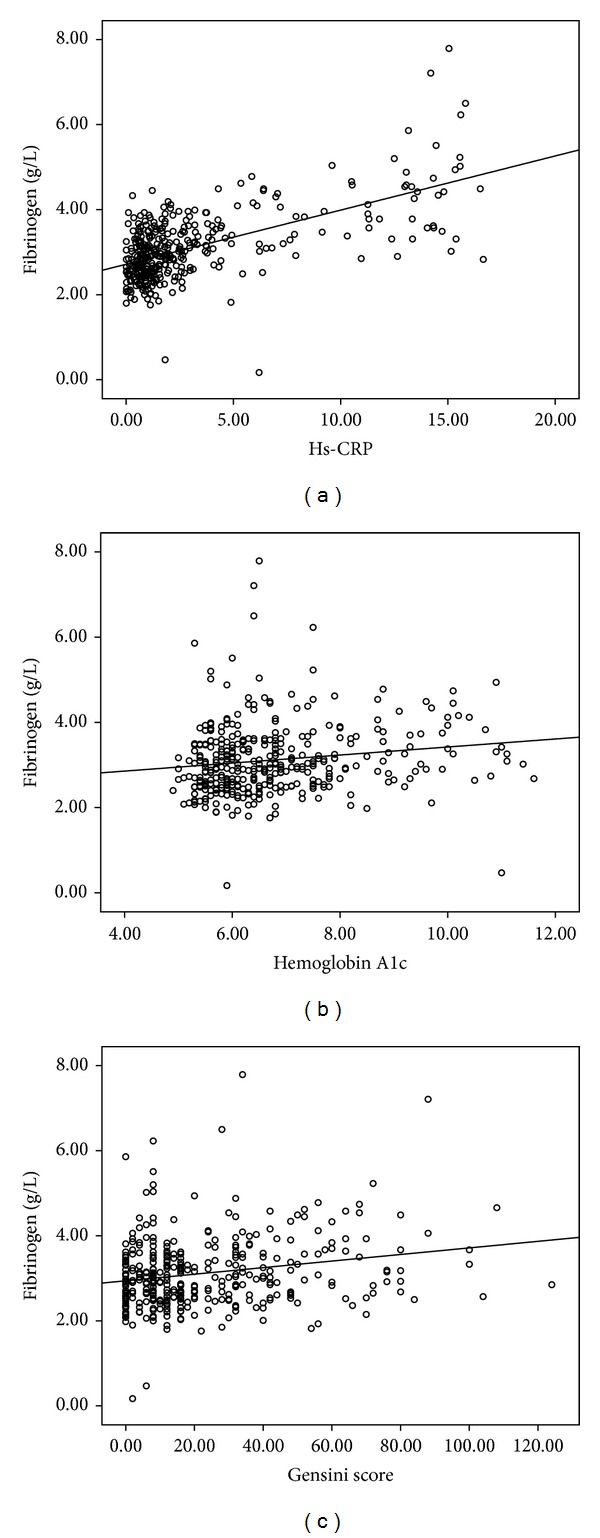
(a) to (c) scatter diagram indicated correlation between plasma fibrinogen and other biomarkers based on Pearson's correlation analysis ((a) hs-CRP; (b) hemoglobin A1c; (c) Gensini scores).

To explore the relationship of plasma fibrinogen concentration and other biomarkers in patients with DM, a correlation evaluation was performed in the present study. Using the Spearman and Pearson correlation analysis, there was definitely correlation among plasma fibrinogen with HbA1c, hs-CRP, and GS (*r* = 0.17, *r* = 0.52, and *r* = 0.21, resp.; all *P* < 0.001; Figures 1(a)–1(c)).

### 3.3. Utility of Fibrinogen for Predicting Severity of CAD in Diabetic Patients

**Figure 2:**
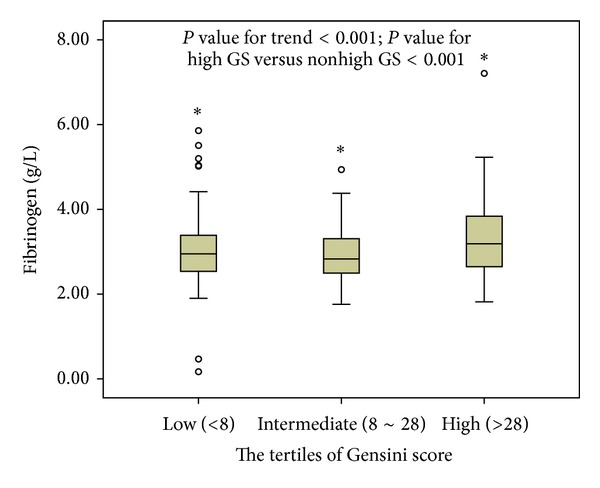
Plasma fibrinogen values according to the Gensini scores.

**Figure 3:**
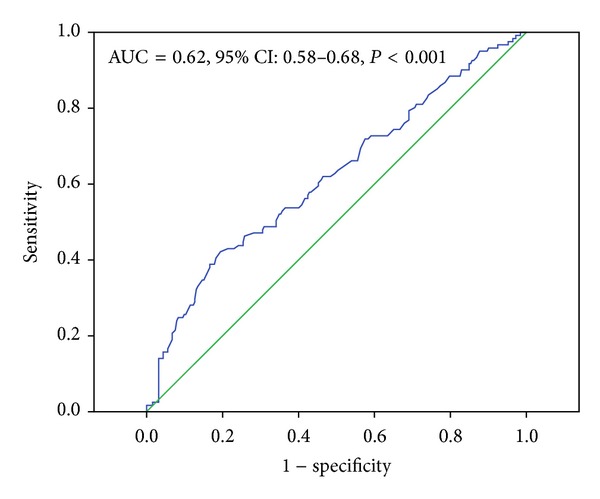
Receiver-operating characteristic (ROC) curves showed discriminatory power of plasma fibrinogen for high Gensini scores.

As shown in Figure 2, there was a statistically significant correlation of plasma fibrinogen concentration with tertiles of GS (chi-squared for trend, *P* < 0.001). Area under the ROC curves also indicated the well discriminatory power of plasma fibrinogen (AUC = 0.62, 95% CI 0.58–0.68, and *P* < 0.001) for CAD (Figure 3). The optimal cut-off value of plasma fibrinogen to predict high GS was 2.5 g/L (sensitivity of 82.6% and 1 − specificity of 73.8%). As indicated in Table 2, the univariate and multivariate logistic regression models with an *r* square of 0.22 suggested that plasma fibrinogen was an independent predictor of the presence and severity of CAD after adjusting for gender, age, BMI, current smoking, hypertension, family history of CAD, hs-CRP, creatinine, and various lipid parameters (OR = 1.40, 95% CI 1.04–1.88, and *P* = 0.026).

## 4. Discussion

The present study prospectively examined the predictive value of plasma fibrinogen concentration with the severity of stable CAD in type 2 diabetic patients. To the best of our knowledge, the present study firstly demonstrated that the plasma fibrinogen concentrations were significantly different assessed by both trend analysis for the tertiles of GS and comparison test for the high and low-intermediate GS groups. In accordance with previous studies on nondiabetic patients, as shown in ROC curves and box bars, our data further suggested that elevated circulating fibrinogen might confer to not only the presence of CAD but also the severity of coronary lesions in diabetic patients with stable CAD. Besides, our findings confirmed that there was correlation of plasma fibrinogen with other systematic inflammatory biomarkers such as HbA1c and hs-CRP (*r* = 0.167 and *r* = 0.520, respectively; all *P* < 0.001). Finally, in the multivariate logistic regression models, plasma fibrinogen was qualified as an independent predictor for the extent of CAD in this cohort. Therefore, the present study suggested an important role of admission plasma fibrinogen in diabetic patients with stable CAD.

Up to date, numerous prospective and retrospective studies have validated the pivotal role of inflammation in the pathogenesis of atherosclerosis [28]. Several clinical cohorts, meta-analysis, and case-control studies have produced compelling evidence that inflammation participates in both initiation and perpetuation of the atherosclerotic process [29–31]. As a short half-life protein and indicator of procoagulant state which was swiftly consumptions, circulating fibrinogen was not only involved in acute phase of ACS but also participated in chronic inflammatory response, which can accelerate the progress of atherosclerosis, and subsequently led to the development of clinical CAD [1, 5, 6, 32–36]. Several lines of evidence revealed the positive association of elevated plasma fibrinogen in peripheral circulation with the prevalence, extent of stable CAD, and acute myocardial infarction on nondiabetic population. Early in 1989, Handa et al. demonstrated that the plasma fibrinogen level was an independent indicator of the severity of coronary atherosclerosis in Japanese estimated both by the numbers of involved vessel and Gensini score [37]. Similar to evidence from the Japanese, this association was also detected among other races such as Italian population [2]. Subsequently, evidence from The ECAT Angina Pectoris Study Group demonstrated the relation of fibrinogen to presence and severity of CAD which was independent of other coexisting heart diseases [38]. Moreover, genetic research suggested that elevated fibrinogen levels were associated not only with the occurrence of CAD but also with more severe CAD, suggesting that measurement of DNA variants of the fibrinogen genes might provide information in predicting CAD severity in addition to that obtained by measuring circulating levels of the relevant clotting factors [39]. Afterwards, date from Tataru et al. indicated the association of fibrinogen with the severity of arteriosclerosis in patients with stable angina pectoris after myocardial infarction [40].

Meanwhile, previous studies have indicated that glycohemoglobin and aging were vital determinants of fibrinogen concentrations in type 2 diabetic patients [3, 11, 41]. Besides, circulating fibrinogen levels were significantly higher in diabetic patients with CAD than those with diabetes or CAD alone [25]. Moreover, study from Rodrigues et al. suggested that the higher circulating fibrinogen levels could predict the progression of coronary artery calcification in type 1 diabetes subjects independent of standard cardiovascular risk factors [14]. Papageorgiou et al. identified that the plasma fibrinogen was a strong predictor of silent myocardial ischemia in diabetic patients, which makes it possible to identify the individuals with high cardiovascular risk [42]. To the best of our knowledge, the present study was the first clinical observation regarding the role of fibrinogen in diabetic patients with stable angina pectoris. The data demonstrated to a positive correlation of fibrinogen concentrations with the severity of CAD.

The exact underlying mechanisms for the present study are unclear. However, the increased levels of plasma fibrinogen were implied not only with the long-term disorder of glycolipid metabolism but also with long time standing of low-grade systematic inflammatory reaction and atherosclerotic plaques progress in these settings [10, 41]. In addition, impaired fibrinolysis or oxidative fibrinogen may exacerbate preexisting CAD and potentiate its evolution [9]. Besides, the formation of thrombi at the site of atherosclerotic lesions plays a central role in atherothrombosis, plaque evolution, and various acute cardiac events [3, 36]. Therefore, fibrinogen might be versatile, which not only played as a biomarker of chronic systematic inflammation and thrombotic disease but also increased risk for premature CAD [7]. Our data partially supported this hypothesis that the higher levels of GS in this specific population were markedly associated with the adverse baseline characteristic such as higher nonspecific biomarkers including HbA1c, hs-CRP, leukocyte counts, uric acid, and fibrinogen. Prior studies have already demonstrated strong correlations of high circulating fibrinogen concentration with above biomarkers [7, 10, 11, 41]. And also, these chemical parameters, either alone or coupled, have been confirmed to be directly involved in the progression of atherosclerotic disease and adverse cardiovascular events. Above all, those findings clearly suggested that the plasma fibrinogen might be very likely to have potential impacts on the vasculature before the establishments of formal diagnosis for clinically overt atherosclerosis disease [8].

## 5. Study Limitations

A cross-sectional observational, single center study with a relatively small sample size may be a limitation. In addition, we did not directly examine the role of plasma fibrinogen in predicting CAD between patients with and without DM. Finally, we failed to track the outcome of study population in the present study.

## 6. Conclusion

Summarily, in the current prospective cohort study, the data clearly suggested that the elevated level of plasma fibrinogen was an independent indicator for the severity of CAD in type 2 diabetic patients. Future investigations may be needed using large sample size for revealing more causative information regarding the role of circulating fibrinogen superimposed on stable CAD of DM.

## Figures and Tables

**Table 1:** Baseline demographic, clinical, and laboratory characteristics based on the tertiles of Gensini scores.

Variables	Low (<8; *n* = 143)	Intermediate (8~28; *n* = 109)	High (>28; *n* = 121)	*P* value for trend^a^	*P* value^b^
Risk factors					
Age, years	56.7 ± 9.9	60.0 ± 9.4	59.8 ± 8.9	0.008	0.121
Male gender	94 (65.7)	78 (71.6)	90 (74.4)	0.291	0.226
BMI (kg/m^2^)	25.7 ± 3.3	24.9 ± 2.8	25.7 ± 3.0	0.120	0.447
Current smoking	68 (47.6)	59 (54.1)	70 (57.9)	0.235	0.121
Hypertension	85 (59.4)	77 (70.6)	82 (67.8)	0.145	0.508
Hyperlipidemia	100 (69.9)	88 (80.7)	99 (81.8)	0.039	0.177
PVD	3 (2.1)	3 (2.8)	2 (1.7)	0.847	0.650
Prior stroke	6 (4.2)	3 (2.8)	6 (5.0)	0.690	0.523
Family history of CAD	7 (4.9)	13 (11.9)	17 (14.0)	0.033	0.064
Laboratory test					
LVEF (%)	62.8 ± 7.7	63.1 ± 7.4	60.2 ± 9.5	0.014	0.003
NT-pro-BNP (fmol/mL)	661.1 ± 486.8	667.9 ± 485.2	893.5 ± 764.8	0.305	<0.001
hs-CRP (mg/L)	3.1 ± 3.9	2.3 ± 3.5	4.0 ± 4.5	0.006	0.006
Leukocyte (10^9^/L)	6.3 ± 1.5	6.2 ± 1.6	6.8 ± 1.5	0.003	0.001
Platelet count (10^9^/L)	204.5 ± 60.4	192.0 ± 45.8	206.5 ± 55.4	0.098	0.224
Fibrinogen (g/L)	3.0 ± 0.8	2.9 ± 0.7	3.3 ± 0.9	0.000	<0.001
D-dimer (mg/dL)	0.4 ± 0.5	0.4 ± 0.5	0.4 ± 0.6	0.075	0.487
Hemoglobin (g/L)	139.4 ± 15.2	138.3 ± 15.6	137.1 ± 15.6	0.505	0.305
HbA1c (%)	6.4 ± 1.2	6.9 ± 1.6	7.0 ± 1.3	0.000	0.004
FBG	5.6 ± 1.6	6.4 ± 2.7	6.2 ± 1.9	0.009	0.253
Bilirubin (umol/L)	15.3 ± 5.4	15.1 ± 5.6	15.4 ± 7.4	0.969	0.836
ALP (IU/L)	64.2 ± 17.9	61.6 ± 19.1	62.6 ± 17.4	0.517	0.816
AST (IU/L)	19.4 ± 13.3	18.5 ± 9.2	17.4 ± 10.0	0.342	0.185
ALT (IU/L)	31.2 ± 33.3	29.7 ± 21.9	28.7 ± 25.1	0.772	0.554
Creatinine	73.8 ± 15.0	75.6 ± 16.4	78.6 ± 14.9	0.041	0.019
Uric acid	335.6 ± 75.6	323.3 ± 80.8	354.6 ± 77.4	0.009	0.005
Lipid profile					
Triglycerides (mmol/L)	1.7 ± 1.0	1.7 ± 0.8	1.8 ± 1.1	0.434	0.230
TC (mmol/L)	4.0 ± 1.0	4.0 ± 0.9	4.1 ± 1.1	0.572	0.360
LDL-C (mmol/L)	2.3 ± 0.9	2.4 ± 0.8	2.5 ± 0.9	0.292	0.121
HDL-C (mmol/L)	1.1 ± 0.3	1.1 ± 0.3	1.0 ± 0.2	0.011	0.009
Lipoprotein (a) (mg/L)	237.7 ± 217.5	190.9 ± 211.2	289.7 ± 283.6	0.008	0.007
apoA (g/L)	1.4 ± 0.3	1.5 ± 0.3	1.4 ± 0.3	0.012	0.057
apoB (g/L)	1.0 ± 0.3	1.0 ± 0.3	1.1 ± 0.3	0.045	0.015
Prior treatment					
Aspirin	136 (95.1)	106 (97.2)	118 (97.5)	0.501	0.463
Beta-blocker	103 (72.0)	87 (79.8)	109 (90.1)	0.001	0.001
ACE-I/ARB	27 (18.9)	22 (20.2)	44 (36.4)	0.002	<0.001
Statin	125 (87.4)	109 (100)	116 (95.9)	0.000	0.258

BMI: body mass index; PVD; peripheral vascular disease; CAD: coronary artery disease; LV-FE: left ventricular ejection fraction; NT-pro-BNP: N-terminal pro-brain natriuretic peptide; hs-CRP: high sensitivity C-reactive protein; HbA1c: glycosylated hemoglobin A1c; FBG: fasting blood glucose; ALP: alkaline phosphatase; AST: aspartate aminotransferase; ALT: alanine aminotransferase; TC: total cholesterol; LDL-C: low density lipoprotein cholesterol; HDL-C: high density lipoprotein cholesterol; ACE-I: angiotensin converting enzyme inhibitors; ARB: angiotensin receptor blocker.

^a^ *P* value obtained from analysis of variance, Kruskal-Wallis test, or chi-squared test; ^b^ *P* value for high GS versus nonhigh (low and intermediate) GS.

**Table 2:** Univariate and multivariate logistic regression analysis to determine the independent predictor of high Gensini Score.

Variables	Univariate		Multivariate		
	O.R. (95% CI)	*P* value	O.R. (95% CI)	*β*	*P* value
Uric acid	1.00 (1.00-1.01)	0.006	1.00 (1.00-1.01)	0.004	0.004
Leukocyte	1.28 (1.10–1.47)	0.001	1.17 (1.00–1.36)	0.16	0.046
LVEF	0.96 (0.93–0.99)	0.004	0.97 (0.95–0.99)	−0.03	0.026
Lipoprotien (a)	1.00 (1.00-1.00)	0.008	1.00 (1.00-1.00)	0.001	0.033
HbA1c	1.24 (1.07–1.44)	0.005	1.24 (1.05–1.46)	0.22	0.009
Fibrinogen	1.69 (1.28–2.23)	<0.001	1.40 (1.04–1.88)	0.34	0.026

LVEF: left ventricular ejection fraction; HbA1c: hemoglobin A1c.
